# An adaptive paradigm for detecting the individual duration of the preparatory period in the choice reaction time task

**DOI:** 10.1371/journal.pone.0273234

**Published:** 2022-09-09

**Authors:** Gurgen Soghoyan, Vladislav Aksiotis, Anna Rusinova, Andriy Myachykov, Alexey Tumyalis

**Affiliations:** 1 Center for Bioelectric Interfaces, Institute for Cognitive Neuroscience, Higher School of Economics University, Moscow, Russian Federation; 2 Department of Psychology, Northumbria University, Newcastle-upon-Tyne, United Kingdom; 3 Center for Cognition and Decision Making, Institute for Cognitive Neuroscience, Higher School of Economics University, Moscow, Russian Federation; Univerity of Texas at Austin, UNITED STATES

## Abstract

According to the sequential stage model, the selection and the execution of a motor response are two distinct independent processes. Here, we propose a new adaptive paradigm for identifying the individual duration of the response preparatory period based on the motor reaction time (RT) data. The results are compared using the paradigm with constant values of the preparatory period. Two groups of participants performed on either an easy (Group 1) or a hard (Group 2) response selection task with two types of stimuli based on the preparatory period parameters: (1) stimuli with a constant preparatory period duration of 0 or 1200 ms and (2) stimuli with adaptive preparatory period durations. Our analysis showed an increase in the duration of the response selection process as a function of increasing task complexity when using both paradigms with constant and adaptive values of the preparatory period duration. We conclude that the adaptive paradigm proposed in the current paper has several important advantages over the constant paradigm in terms of measuring the response accuracy while being equally efficiently in capturing other critical response parameters.

## Introduction

Speed and accuracy of a person’s reactions in a constantly changing environment is critical for adaptation. In some situations, events are unpredictable, and an individual’s response may take time to initiate and its results may be prone to error. In other situations, responses may be quite accurate and rapid since a person may be able to predict the outcomes of the event and, therefore, prepare their responses in advance. In this study, we propose and test an adaptive paradigm for measuring the duration of the motor response preparation period allowing its accurate estimation and leading to a better understanding of the cognitive mechanisms of motor control.

Psychological research often employs Choice Reaction times (CRTs) to investigate response selection processes. According to the Sequential Stage Model (SSM), a motor response is a forward process that comprises independent stages of sensory analysis, response selection, and motor execution [1]. The sensory and the motor stages carry out information processing in parallel and therefore their activation can overlap in time in the case of serial reactions. By contrast, the response selection stage is sequential resulting in a structural [1, 2] or strategic [3] information flow ’’bottleneck’’, although this sequential processing principle may be violated in boundary conditions [4].

The independence assumption regarding the selection and the execution of a response allows to replace the response selection process with the period (often called “foreperiod”, FP) between the presentation of a preliminary warning signal or a response direction cue and an imperative go signal, prompting the selected response execution [5]. A long FP leads to the emergence of a complex effect including a decrease in the response latency and an increase in its accuracy [6–9] as well as a decrease in the sensory thresholds of the response-relevant features of the imperative signal [7, 10]. Korolczuk et al [11] investigated FP-bound mechanisms of response preparation and found an increase in general response inhibition and selected response facilitation. This preparatory process reduces the motor program conflict, and the response is therefore executed as a simple process similarly to a prepared reflex [12]. Unfortunately, interpretation of the data when using constant FP durations is somewhat problematic, because short FP leave the reaction selection process unfinished and long FP periods include an additional response inhibition process [13, 14].

However, in a series of studies, Immink & Wright [15, 16] investigated the effects of the task dependent FP length. The authors used a self-select paradigm wherein participants examine experimental stimuli for as long as they need before providing a response. This stimulus examination period is termed Study Time (ST). When response is fully prepared, the participant presses a key and then an imperative signal is presented after a variable delay. One common finding from these studies is that ST duration increased with random stimuli presentation compared to a blocked stimuli presentation [15]. Also, ST duration increases for complex responses compared to simple responses [16]. However, both RTs and STs for random stimuli presentation were associated with a greater decrease in magnitude compared to the blocked stimuli presentation following intense training [17]. The interpretation of the experimental findings in this paradigm is problematic because the paradigm does not allow for separating response selection processes of the primary task from the decision-making processes of the response selection completeness. It also includes a waiting period for the imperative signal leading to an increase in the working memory load and affecting the RTs.

Arguably, the most influential response selection model is The Diffusion Decision Model (DDM) [18]. According to this model, CRT is a summation product of non-decision and decision processes. Non-decision process includes the sensory analysis and the motor response execution period. The decision processes are conceptualized as accumulating noisy evidence until the decision threshold is reached. DDM assumes that this process is stochastic in nature and its duration depends on a set of parameters—growth rate, starting point, and boundary separation. Note that estimating parameter values in DDM depends on strong assumptions about the values’ distribution [19] and, as such, it is problematic with regard to the prediction of the significant RT parameters [20]. A simpler version of DDM is a Linear Ballistic Accumulation (LBA) model [21], which assumes a linear and predictable evidence accumulation process for as many accumulators as there are response options. As a result, a response whose decision process reaches the response threshold earlier will have a higher chance of being executed.

Despite their high predictive power regarding the response accuracy and latency, DDM and LBA also have certain limitations in their ability to estimate the two-step decision-making process. In the study by Sun & Landy [22] participants performed on a modified sensory decision-making task. Participants were first presented with visual stimuli, and then they heard a sound signal at constant periods signaling the execution of a motor response. Based on the data, the authors proposed a two-stage sensory decision model. At the first stage, the assessment of the sensory stimulus takes place–a stochastic process of evidence accumulation. This leads to a displacement of the starting point for the beginning of the second process, which is linear and deterministic, and it reflects the specific response selection. The authors show that this model predict results significantly better than the classical DDM.

Another two-stage paradigm is the Compelling Saccade paradigm [23]. The sequence of task events, however, is reversed–first, a go signal is presented and then a cue. At the first stage, a stochastic process takes place, directed to one of the sides, therefore shifting the starting point. Then, the cue is presented at the second step, and if the location of stochastic process and the cue direction match, then the growth rate increases; if they do not match, then the growth rate decreases. Depending on the period length, the first process can be more or less proximal to the border, thus the reversal of the trajectory of the second process is more or less successful. Importantly, this model also predicts results significantly better than the classical DDM.

Finally, research by Servant et al. [24] proposed a Dual-Threshold Diffusion Model, according to which the reaction time period is divided into premotor and motor periods based on the EMG latency. The results showed that decision making in the situation of increasing uncertainty using a random dot motion task causes an increase in the duration of both periods. Importantly, the authors found high correlations (r>0.95) between the duration of the premotor and motor periods between participants’ responses and low correlations (r<0.13) within participants’ responses. At the same time, there were high correlations between classical DM parameters and DTDM parameters considered as an extension of DM with two borders.

Here, we propose and test a novel adaptive paradigm allowing examination of the FP duration more directly. Following previous reports [6–9, 15, 16], the length of the preparatory period, measured as the gap between the cue and the imperative go signal, should significantly affect the subsequent response RT magnitude. Thus, moving in opposite direction estimating the FP length can be done by examining the RT data. Experimentally, we can proportionally change the duration of the FP in the trial *n+1* based on the RT in the trial *n*. Importantly, our adaptive paradigm can calculate response parameters online. As such, it allows adjusting them individually or in accordance with the task parameters. It also allows manipulating the task flow online and in the necessary direction, tightening or weakening the requirements, skewing online data calculation, or biasing the response selection process in the predicted direction.

Unlike constant FP durations, this affords analysis of the individual FP length values. In a recent review, Klapp and Maslovat [25] note that the motor system is tuned to provide a motor response period including a short delay period aiming at preventing premature responses. When using long constant FPs, the selection process endpoint shifts the go process closer to the response threshold, but it also requires a delay until the presentation of imperative signal, thus, activate an additional reaction inhibition process. An efficient balance between the activation and the inhibition processes close to the threshold may be upset and it may lead to faster responses to any distractor stimuli, as predicted by the prepared reflex approach [26]. The proposed adaptive paradigm does not require a waiting period for the imperative go stimulus because the response execution process follows the response selection process in a timely and congruent fashion thus reducing potentially premature or erroneous responses.

In the current study we used constant FP durations, indicating the boundary conditions of fully prepared and unprepared responses and additionally we manipulated response selection complexity in order to analyze FP and RT changes. This manipulation concerns only the correspondence rule between the stimuli and responses but not the motor response complexity. We expected to replicate two well-documented effects: (1) an RT decrease with longer constant preparatory periods and (2) an RT increase with increasing task complexity.

For adaptive task, we assume the following effects: (1) the adaptive foreperiod length will be longer for the hard task compared to the easy task, condition, reflecting a complex response selection process and (2) the adaptive RT difference between the hard and the easy tasks will be non-significant since the response execution process the same for both tasks.

## Materials and methods

### Participants

67 participants were randomly assigned to one of the two experimental groups–an easy or a hard version of the CRT task. The study used a group design to reduce the influence of the learning factor. In the hard-task group, the data from two participants were excluded from the analysis due to containing more than 40% of missing responses to a stimulus with the unprepared response, one participant made more than 40% of premature responses to stimuli with the long constant foreperiod and one participant’s data were low in response accuracy for all types of stimuli. Data from two participants in the easy-task group were also removed from analysis due to a high proportion of RT values outside two standard deviations from the mean. As a result, the data from 30 participants in the easy-task group (Mean ± SD, age = 21.2 ± 2.25 years, males = 14, education = 14.3 ± 2.04 years) and 31 participants–in the hard-task group (Mean ± SD, age = 21.39 ± 2.40 years, males = 13, education = 14.52 ± 2.39 years) were submitted for statistical analysis. The groups did not differ in age (t (59) = 0.31, p = 0.755), sex ratio (*χ*^2^ (df = 1) = 0.14, p = 0.710), and education level measured as the number of years in formal education (t (59) = 0.32, p = 0.750).

All participants were students or staff of Higher School of Economics with no self-reported history of neurological or psychological impairments. Participation was voluntarily, and an individual informed consent was obtained from each participant. Participants were included in the study if the duration of sleep on the eve of the study exceeded 6 hours, if there was no alcohol intake during the previous day, and if they did not consume tonic drinks for at least two hours prior to the experimental session. The study was approved by the Ethics Committee of the Higher School of Economics, and it was conducted in accordance with the Declaration of Helsinki.

### Procedure

Participants completed the task individually in a room with controlled lighting, sitting in a chair at the table in front of a computer monitor at the viewing distance of approximately 60 cm. Participants first filled out a demographic questionnaire, and then they completed the main experimental task. Finally, they were debriefed at the end of the session. Experimental instructions were presented in the center of the screen prior to the task. Participants were instructed to produce their responses as fast and as accurately as possible. If the participant had further clarification questions, these were answered by the experimenter verbally. The time of familiarization with the instructions was controlled by the participants.

**Fig 1:**
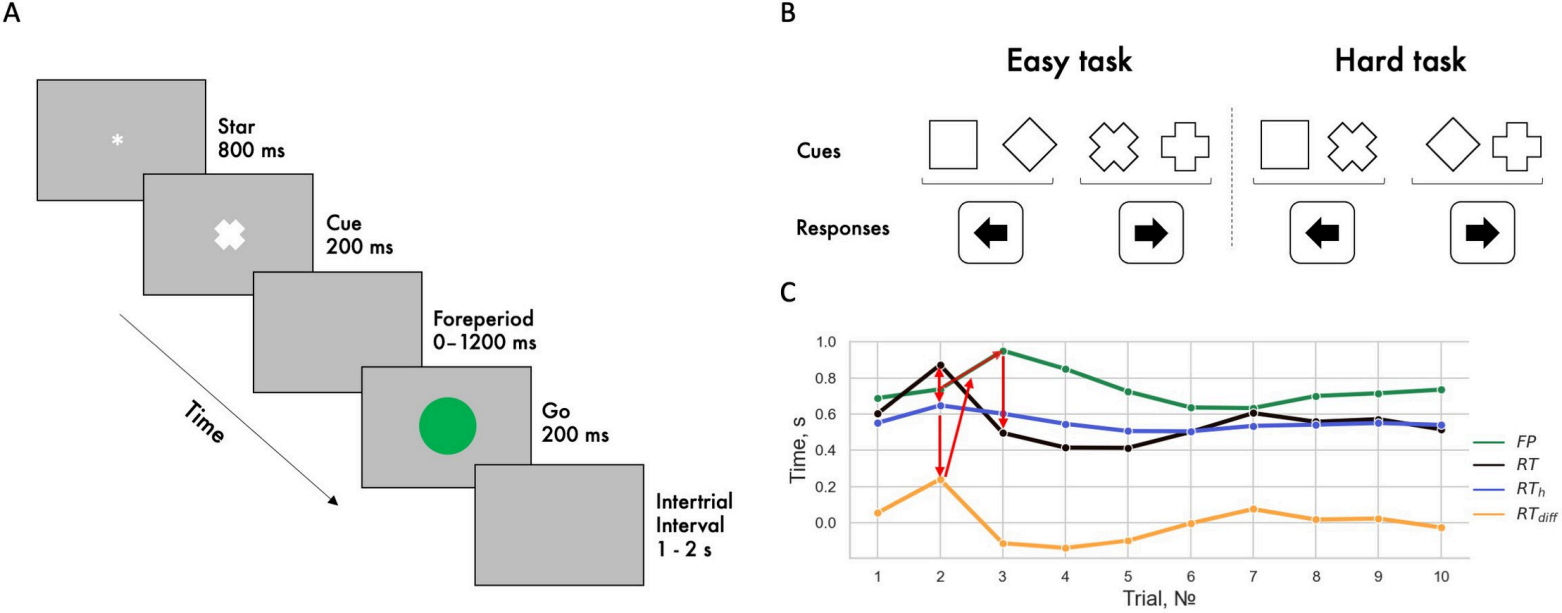
Experimental trial sequence. *Note*. A. Time course of events during an experimental trial. B. Cue and response correspondence for the easy and the hard tasks. C. Illustration of parameter computation for the adaptive method. Data were taken from one participant who responded to ten consecutive stimuli. Red arrows indicate the event sequence in two trials. First, the difference between RT and RTh is calculated, then the FP is increased as a function of this difference leading to the RT decrease in the subsequent trial.

During the experimental trial, first, an asterisk was presented for 800 ms, then a cue for 200 ms. This was followed by a foreperiod of varying duration and a go stimulus for 200 ms. Upon presentation of the go signal, the participants performed a motor response by pressing the left or right arrow on the keyboard. The waiting time for a response was 1400 ms. The intertrial interval was randomized from 1000 to 2000 ms (Fig 1A). The cues-to-response correspondence rule was as follows: (1) for the hard version of the task, a square or a rotated cross was associated with pressing the left keyboard arrow; a rotated square or a cross were associated with pressing the right keyboard arrow, (2) for the easy version of the task, the square was associated with the left keyboard arrow, and the cross–with the right keyboard arrow, regardless of the angle of the stimulus rotation (Fig 1B).

In the present study, the foreperiod was of two categories—constant and adaptive. Constant FP durations were set at 0 and 1200 ms (RT_FP0_ and RT_FP1.2_) and it was the boundary conditions of unprepared and fully prepared responses (Thomaschke et al., 2011b; Shin & Proctor, 2018). For the stimuli with the adaptive FP, its length was updated in a trial-by-trial fashion based on the RT (FP_ad_ and RT_ad_) in accordance with the following formula:

$$FP_{n+1}=FP_{n}+k\times (RT_{n}‐RT_{h})$$ (1)

where FP_n+1_ is the FP duration in n+1 trial, FP_n_ is the FP duration in n trial, RT_n_ is the RT in trial n, and RT_h_ is the RT history, k is a scaling coefficient. This formula is an alpha-beta filter for smoothing FP prediction values.

The Formula (1) is based on the following consideration. Since RT depends on FP and in order to identify the individual duration of these variables, we bound the changes in the FP duration to the values of the RT deviation in the current trial from the exponentially smoothed RTs of the previous trials. Thus, the FP duration change follows the change in RT. Since FP and RT are negatively related, i.e., a longer FP duration leads to a shorter RT, then this is a negative feedback system.

The use of the scaling *k* parameter is based on three reasons. First, the immediate response history affects the RT [27–29]. Second, the response time includes motor and program noise [30]. Third, attentional fluctuations affect RTs [31]. Via the use of an exponential smoothing, the influence of these factors is reduced, and we can obtain a more stable indicator of the individual RT values.

To improve stability, we have reduced the contribution of the deviation of RT_n_ from RT_h_ to FP_n+1_ by using the scaling coefficient k = 0.75. The k value was selected experimentally during preliminary testing of the paradigm on several participants.

Following the calculation of the FP length, the RT history parameter was updated according to the following formula:

$$RT_{h+1}=w\times RT_{h}+(1‐w)\times RT_{n}=RT_{n}‐w\times (RT_{n}‐RT_{h})$$ (2)

where RT_h+1_ is a weighted moving average of the response latency history (RT_h_), the RT_n_ in the trial n, and weighting coefficient w = 0.7. The Formula 2 represents the low-pass filter of the RT data.

With this calculation logic, the change in the reaction-time history variable becomes smoother. A smaller bias of the averaging towards RT_h_ leads to larger changes in RT_h+1_, considering a greater influence of RT_n_. Consequently, in the n + 1 trail, the deviation of RT_n_ from RT_h_ can be either very small or very large rendering FP unstable (see Formula 1). On the other hand, a greater bias in averaging towards RT_h_ should lead to smaller changes in RT_h+1_ and the system should become overly rigid and unable to adaptively change. The value of w = 0.7 was also selected based on a preliminary test of the experimental paradigm.

The initial values of RT_h_ and FP_n_ were set at 0.4 s and were then updated after each trial. If a preliminary response or no response were registered in a given trial, the RT_h_ and FP_n_ were left unchanged.

Fig 1C shows the effect of the algorithm for changing the FP length depending on the deviation of the response time from the RT_h_. The figure portrays an excerpt from successive responses to stimuli of one of the participants: When the RT deviates from RT_h_ in the positive direction, the FP length increases in the next presentation, following a RT decrease as result of FP effect and gradual stabilization of the RT and, accordingly, the FP lengths.

### Stimuli

The stimuli were presented on a computer screen centrally against a gray background. They were of two categories—cues and go signals. A square and a cross were used as cues, presented with a rotation angle of 0° or 45°. The cues were white, 2.8 cm wide, and had an angular size of 2°40’ with a viewing distance of 60 cm. The go signal was a green circle, 5.5 cm in diameter and having an angular size of 5°15’ with a viewing distance of 60 cm.

Experimental trials were presented in individually randomized sequences in a single block. A total of 120 stimuli were presented– 40 trials per each category. Before completing the main block, the participants performed a training session consisting of 24 trials. Practice session was different from the main experimental part in terms of the presence of a cue regarding the connection rule between stimuli and responses presented at the bottom of the screen and a feedback during 500 ms regarding the correctness or an absence of a response after each trial.

The experiment was carried out on a PsychoPy3 (release 2020.2.10) software [32].

### Data processing

The RT and accuracy data were preprocessed separately for each category. Data preprocessing included the following steps. First, all trials with premature responses were removed. A response was considered premature if it occurred during the period after the cue onset and before the go signal onset. Since there was no such period for the RT_FP0_ stimuli, the number of premature responses was calculated only for the RT_ad_ and RT_FP1.2_ stimuli. Second, the number of trials with no response was calculated. Third, trials with extremely early/anticipatory responses, RT<100 ms, were removed. RTs for the remaining trials were averaged for each participant and stimulus category.

### Data analysis

Mean RTs for RT_FP0_ and RT_FP1.2_ trials, as well as mean RT_ad_ values and mean FP_ad_ values for the hard- and easy-task groups were subjected to mixed ANOVA analyses with Group as the between-participant factor (Easy task, Hard task) and two within-participant factors: FP type (Constant, Adaptive) and FP location (In, Out). The two levels of the FP location factor indicate that the preparatory period is included in the response execution period, that its duration is estimated (level In for the RT_FP0_ and FP_ad_), or that the preparatory period falls outside of the RT period (level Out for the RT_ad_ and RT_FP1.2_).

The sphericity was corrected using the Greenhouse-Geyser criterion, the effect size was estimated by the partial eta squared. Multiple comparisons were made with Bonferroni- corrected p-values. The relationship between the variables was assessed by the Pearson correlation coefficient. Post-hoc power analysis was conducted in G-Power 3.1 software. The significance level was set at 0.05.

## Results

### Reaction times

Analysis of the mean RTs for the RT_FP0_, RT_ad_, and RT_FP1.2_ stimuli was carried out as follows. First, we examined the ANOVA effects using the stimuli with constant FPs to establish the effectiveness of the manipulation. Following this, the main analysis was carried out, which included an additional factor FP type, and it aimed at comparing the results obtained using the constant and the adaptive FP durations. Finally, we performed separate ANOVAs to clarify and localize the registered effects.

#### Manipulation check

First, we examined the Group effect (Easy, Hard) on boundary stimuli, i.e., stimuli with the period duration between the cue and the go signal equal to 0 and 1.2 s. This analysis should confirm the effectiveness of the experimental manipulation regarding task complexity as well as the effect of the preparatory period on the RTs. We expected a reliable Group effect manifested as an RT increase in the hard-task group. A reliable Stimulus effect was also expected expressed as an RT decrease as a function of the increase in the foreperiod duration. Finally, we predicted Stimulus x Group interaction since long preparation should reduce the RT difference between the groups.

**Fig 2:**
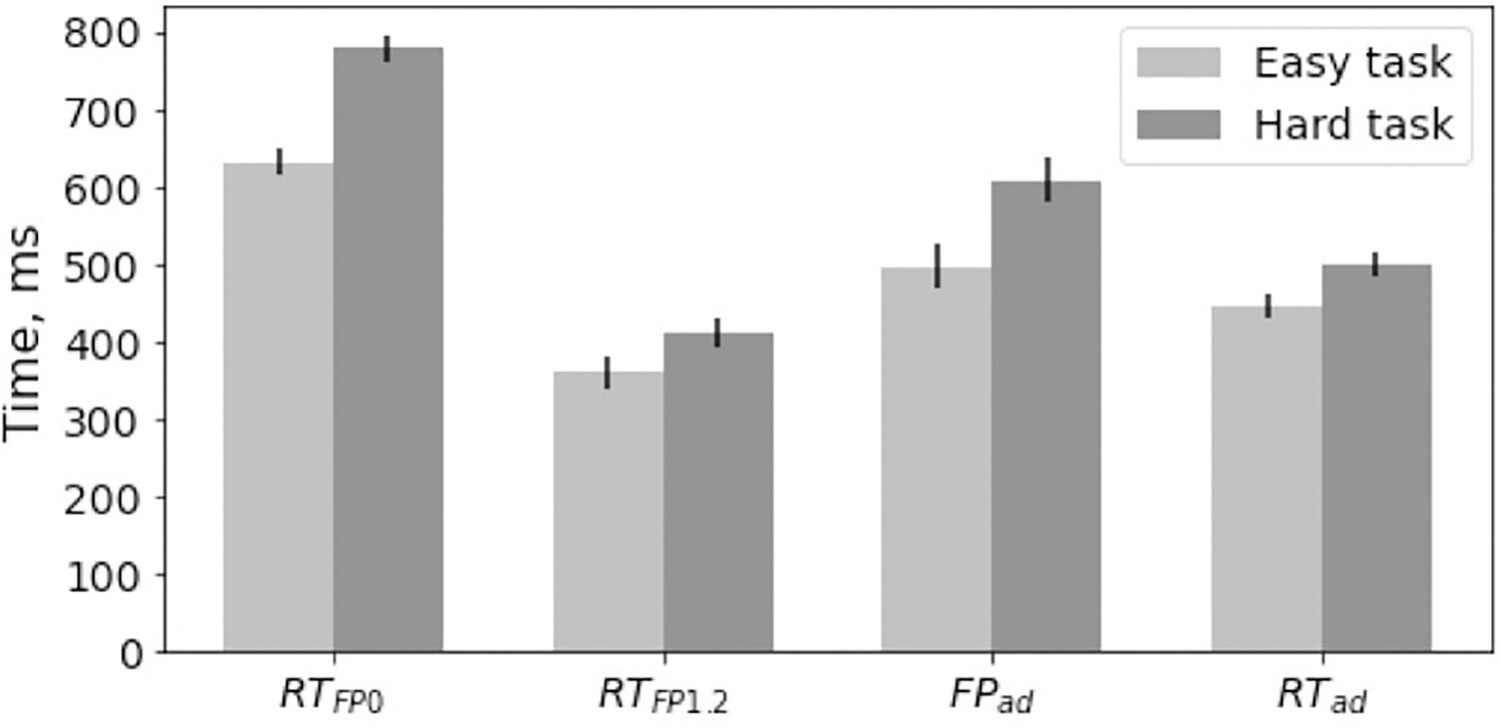
Mean and standard errors for RT_FP0_, RT_FP1.2_, FP_ad_ and RT_ad_ in easy and hard task.

Fig 2 depicts mean RT values and standard errors for all stimuli categories for both experimental groups. A mixed ANOVA with Group (Easy, Hard) and Stimulus (RT_FP0_, RT_FP1.2_) as independent factors revealed a reliable main effects of Group (F(1, 59) = 16.84, p < 0.001, $\eta_{p}^{2}$ = 0.22) and Stimulus (F(1, 59) = 842.29, p < 0.001, $\eta_{p}^{2}$ = 0.93) as well as a Group x Stimulus interaction (F(1, 59) = 19.78, p < 0.001, $\eta_{p}^{2}$ = 0.25). A reliable Group effect indicates an RT increase in the hard-task group (595 ± 17 ms) compared to the easy-task group (496 ± 17 ms). A reliable Stimulus effect indicates an RT decrease for RT_FP1.2_ (387 ± 15 ms) compared to RT_FP0_ (704 ± 12 ms). The interaction between the two factors suggests that the response time decreased in both groups for stimuli RT_FP0_ and RT_FP1.2_ (Easy task: RT_FP0_ = 631 ± 17 ms, RT_FP1.2_ = 362 ± 21 ms, t (29) = 24.58, p < 0.001 Hard task: RT_FP0_ = 778 ± 16 ms, RT_FP1.2_ = 411 ± 20 ms, t (30) = 19.54, p < 0.001. Between-group comparisons confirmed that the response time was significantly longer in the hard-task group for RT_FP0_ compared to the easy-task group (t (59) = 6.29, p < 0.001) while the between group contrast for RT_FP1.2_ was unreliable (t (59) = 1.71, p = 0.093).

Thus, our RT analysis showed that the experimental manipulation was effective and that it resulted in an increase in response latencies as a function of an increase in the task complexity (i.e., the complexity effect) as well as a decrease in response latencies as a function of an increase in the foreperiod duration (i.e., the preparation effect). At the same time, group RT differences with a long preparation period were not reliably different indicating the registration of a fully prepared response in both cases. Therefore, we can conclude that the RT differences between the easy-task and the hard-task groups for the RT_FP0_ stimuli reflect the difference in the duration of the response selection period.

#### Main analysis

Note that the main goal of this study was to assess the novel adaptive paradigm by comparing results obtained with constant and adaptive FPs. To this end, we conducted a mixed ANOVA with the factors Group (Easy task, Hard task), Foreperiod Location (In, Out), and Foreperiod Type (Constant, Adaptive). The Foreperiod Location factor included *IN* levels with response selection period (RT_FP0_ and FP_ad_), and *OUT* levels–for the responses to the stimuli, where the preparation period was outside of the response period (RT_ad_ and RT_FP1.2_). This analysis returned a reliable main effects of Group (F (1, 59) = 10.91, p = 0.002, $\eta_{p}^{2}$ = 0.16), Foreperiod Type (F (1, 59) = 20.68, p < 0.001, $\eta_{p}^{2}$ = 0.26), and Foreperiod Location (F (1,59) = 1077.31, p < 0.001, $\eta_{p}^{2}$ = 0.95) as well as two two-way interactions Foreperiod Location × Group (F (1, 59) = 41.88, p < 0.001, $\eta_{p}^{2}$ = 0.42) and Foreperiod Location × Foreperiod Type (F (1, 59) = 182.14, p < 0.001, $\eta_{p}^{2}$ = 0.76). For all the observed effects ε was equal to 1. The Foreperiod Type × Group (F (1, 59) = 1.18, p = 0.283, $\eta_{p}^{2}$ = 0.02) and the three-way interaction Foreperiod Type × Foreperiod Location × Group (F (1, 59) = 1.16, p = 0.285, $\eta_{p}^{2}$ = 0.02) were not reliable.

Response times were longer in the hard-task group (575 ± 19 ms) compared to the easy-task group (484 ± 20 ms). Furthermore, unprepared responses were longer (Foreperiod Location (In): 629 ± 16 ms) compared to prepared responses (Foreperiod Location (Out): 431 ± 12 ms). The interaction between these two factors indicates that the RT decrease was more substantial in the hard-task group (Foreperiod Location (In) = 694 ± 22 ms; Foreperiod Location (Out) = 457 ± 17 ms; t (61) = 11.61, p < 0.001) compared to the easy-task group (Foreperiod Location (In) = 564 ± 22 ms; Foreperiod Location (Out) = 405 ± 18 ms; t (59) = 9.46, p < 0.001). When the preparation period was inside the response period, the differences between the groups were larger (Foreperiod Location (In): t (120) = 4.64, p < 0.001) compared to the trials when the preparation period was outside of the response period (Foreperiod Location (Out): t (120) = 2.63, p = 0.010, nonsignificant after Bonferroni correction).

The difference between the FP types was in the longer response times when using the constant FP durations (546 ± 12 ms) compared to the adaptive (514 ± 16 ms) FP durations. This effect depended on the FP location. For the constant FP, the differences in RT between the stimuli were greater (RT_FP0_ = 704 ± 12 ms, RT_FP1.2_ = 387 ± 15 ms see Manipulation check) compared to the adaptive FP and RT (FP_ad_ = 554 ± 21 ms, RT_ad_ = 474 ± 11 ms, t (60) = 7.19, p < 0.001). Also, response times for RT_FP0_ were longer than FP_ad_ (t (60) = 10.68, p < 0.001), but response times for RT_FP1.2_ were shorter than RT_ad_ (t (60) = -12.20, p < 0.001).

Importantly, the Group × Foreperiod Type interaction as well as the three-way interaction were unreliable; i.e., the Group effect and the interactions between the Group and the Foreperiod Location were independent of the Foreperiod Type. Thus, the adaptive FP captured the same basic selection and response execution mechanisms that are implemented in the paradigm with constant values of the preparatory period duration.

We also performed a separate ANOVAs for FP_ad_ vs RT_ad_, RT_FP0_ vs FP_ad_, and RT_ad_ vs RT_FP1.2_. For the adaptive stimuli, the mixed ANOVA included Period (FP_ad_, RT_ad_) and Group (Easy task, Hard task) factors. Significant main effects of Group (F (1, 59) = 6.73, p = 0.012, $\eta_{p}^{2}$ = 0.10) and Period (F (1, 59) = 57.91, p < 0.001, $\eta_{p}^{2}$ = 0.50) as well as Group × Period interaction (F (1, 59) = 8.05, p = 0.006, $\eta_{p}^{2}$ = 0.12) were registered. The observed Group effect revealed an RT inflation in the hard-task group (556 ± 23 ms) compared to the easy-task group (473 ± 23 ms). The observed Period effect indicates that the foreperiod duration was longer (554 ± 21 ms) compared to the RT (474 ± 11 ms). The interaction between these two factors suggests that the foreperiod was longer than the RT in Hard task group (FP = 610 ± 30 ms, RT = 501 ± 15 ms, t (30) = 7.22, p < 0.001), but not in Easy task group (FP = 497 ± 30 ms, RT = 448 ± 16 ms, t (29) = 3.47, p = 0.020, nonsignificant after Bonferroni-correction). At the same time, the difference between the groups was reliable for the foreperiod length (t (59) = 2.66, p = 0.010), and it did not reach the significance threshold for the RTs (t (59) = 2.45, p = 0.017, unreliable after Bonferroni-corrected critical p = .0125). These findings replicate our RT data for constant paradigm.

Several conclusions can be drawn from our analyses. First, an increase in the task complexity led to an increase in the preparatory period duration, which can be considered as the task verification. Second, the adaptive paradigm effects replicated the effects of the constant-periods paradigm indicating that the adaptive paradigm captured the same response preparation and execution processes as the constant FP values paradigm. These paradigms differ only in the extracted effects’ magnitudes whereby the difference between unprepared and prepared responses when using the constant paradigm were greater than the differences between the FP and RT durations–when using the adaptive paradigm.

Additionally, we examined the effects of gender and age on RT and FP data. While gender was found to influence the reaction time values (Males = 485 ± 19 ms, Females = 566 ± 17 ms, F (1, 57) = 9.90, p = 0.003, $\eta_{p}^{2}$ = 0.15), it did not interact with any other factors in the main analysis. To analyze the influence of the participants’ age, we divided the sample by means of a median split into two groups. An ANOVA performed with an additional age factor showed a reliable main effect of Age (Younger = 504 ± 18 ms, Older = 562 ± 20 ms, F (1, 57) = 4.81, p = 0.032, $\eta_{p}^{2}$ = 0.08), which did not interact with any other factors.

### Correlations

According to the preparation effect, the FP length increase should be accompanied by the RT decrease. Is this pattern preserved when changing the duration of the preparatory period using the adaptive method? To answer this question, we conducted a correlation analysis between the preparation period duration values and the RTs in the trials with adaptive FP changes for each participant in both groups. This analysis showed that the correlations between FP_ad_ and RT_ad_ ranged from -0.48 to 0.20 in the easy-task group and from -0.43 to 0.34 –in the hard-task group. To examine the relationship between these variables, the squares of the correlations, the coefficients of determination, were calculated reflecting the percentage of the common variance for the two variables. The proportion of the explained variance did not differ between groups (Easy task: 4.4%; Hard task: 6.8%; t (59) = 1.75, p = 0.084). Thus, the mean values were small and, considering the correlation direction, the relationship should be close to zero. Hence, we suggest the independence of the processes of preparation and execution of the motor response.

Between individual correlations of mean values for RT_FP0_, RT_FP1.2_, FP_ad_ and RT_ad_ in the easy and hard-task groups are presented in Table 1 and show the sharp contrast with the within individual correlations.

**Table 1:** Correlations between RTs for stimuli with constant and adaptive foreperiods in groups with easy and hard tasks.

	RT_FP0_	RT_FP1.2_	FP_ad_	RT_ad_
**RT**_**FP0**_	-	0.85 (<0.001)*	0.89 (<0.001)*	0.90 (<0.001)*
**RT**_**FP1.2**_	0.52 (0.05)	-	0.93 (<0.001)	0.93 (<0.001)
**FP**_**ad**_	0.70 (<0.001)	0.86 (<0.001)	-	1.00 (<0.001)^#^
**RT**_**ad**_	0.70 (<0.001)	0.84 (<0.001)	0.99 (<0.001)^#^	-

*—significant (p < 0.05) differences between groups

#—significant (p < 0.05) differences between variables within groups

*Note*. The upper triangle is an easy-task group, the lower triangle is a hard task group. The level of significance is indicated in parentheses.

First, the table shows that all correlations were reliable and positive. The RTs for the adaptive FP shows high correlations with the RTs for the constant FP, i.e., it captures the same process.

Second, the correlations between RT_FP0_ and the rest of the variables were greater in the easy-task group than in the hard-task group. This reflects an additional variability in the data due to an increase in the task complexity. Third, within each group, the correlations between FP_ad_ and RT_ad_ were greater than the other correlations, which reflects that adaptive FP captures a larger variability of the preparation and response execution times in comparison to the constant FP method. This means that the same process is measured by the two variables. Therefore, it is likely to reflect individual dynamics of the response selection and execution, then task construction.

### Task accuracy

#### Number of preliminary responses

A mixed ANOVA with Group (Easy, Hard) and Stimulus Type (RT_FP1.2_, RT_ad_) factors revealed a reliable main effects of Group (F (1, 59) = 5.51, p = 0.022, $\eta_{p}^{2}$ = 0.09) and Stimulus Type (F (1, 59) = 65.55, p < 0.001, $\eta_{p}^{2}$ = 0.53) as well as Stimulus Type x Group interaction (F (1, 59) = 4.91, p = 0.021, $\eta_{p}^{2}$ = 0.08). The reliable Group effect showed a greater number of premature responses for the Hard task (7.10 ± 0.89%) compared to the Easy task (4.12 ± 0.90%) and Stimulus Type effect revealed a greater number of premature responses for the constant FP stimuli (10.49 ± 1.21%) compared with the adaptive FP stimuli (0.74 ± 0.26%).

For RT_ad_, the number of premature responses was low in both groups (Easy task: M = 0.58%; Hard task: M = 0.89%, t(59) = 0.59, p = 0.559). For stimuli RT_FP1.2_, there were more premature responses in the hard-task group compared to easy-task group (Easy task: M = 7.67%; Hard task: M = 13.31%, t(59) = 2.33, p = 0.023, but nonsignificant following Bonferroni correction). For both Easy and Hard tasks, the number of premature responses were higher for RT_FP1.2_ compared with RT_ad_ (Easy task: t (29) = 5.41, p < 0.001; Hard task: t (30) = 6.20, p < 0.001).

#### Number of missing responses

A mixed ANOVA with Group (Easy, Hard) and Stimulus Type (RT_FP0_, RT_FP1.2_, RT_ad_) factors revealed a reliable main effects of Group (F (1, 59) = 8.31, p = 0.006, $\eta_{p}^{2}$ = 0.12) and Stimulus Type (F (1, 59) = 11.55, p < 0.001, $\eta_{p}^{2}$ = 0.16) as well as Stimulus Type x Group interaction (F (1, 59) = 7.44, p = 0.001, $\eta_{p}^{2}$ = 0.11). Between group comparisons show a greater number of missing responses for Hard task (6.94 ± 0.93%) then for Easy task (3.11 ± 0.95%). Comparisons between groups for each stimuli type revealed a greater number of missing responses for Hard task for RT_FP0_ (Easy task group: 3.58 ± 1.75%; Hard task group: 11.69 ± 1.72%, t (59) = 3.30, p = 0.002) but not for RT_FP1.2_ (Easy task group: 3.50 ± 0.95%; Hard task group: 4.92 ± 0.94%, t (59) = 1.06, p = 0.292) and RT_ad_ (Easy task group: 2.25 ± 0.78%; Hard task group: 4.19 ± 0.77%, t (59) = 1.78, p = 0.080).

Comparisons between stimuli types revealed a greater number of missing responses for RT_FP0_ (7.64 ± 1.23%) compared to both RT_ad_ (3.22 ± 0.55%, t (60) = 4.07, p < 0.001) and RT_FP1.2_ (4.21 ± 0.67%, t (60) = 2.88, p = 0.006). RT_ad_ and RT_FP1.2_ did not differ from each other (t (60) = 1.52, p = 0.133). Within-group comparisons revealed reliable main effects of Stimuli Type for Hard task group (F (2, 60) = 13.03, p < 0.001, ε = 0.69, $\eta_{p}^{2}$ = 0.30) but not for Easy task group (F (2, 58) = 1.07, p = 0.348, ε = 0.82, $\eta_{p}^{2}$ = 0.04). Comparisons within Hard task group the revealed there were more missing responses for RT_FP0_ than for both RT_ad_ (t (30) = 4.08, p < 0.001) and RT_FP1.2_ (t (30) = 3.56, p = 0.001).

Thus, the hard-task group showed a larger response attrition for RT_FP0_ compared to the easy-task group and more premature responses for RT_FP1.2_. The lack of responses may be reflect the fact that the duration of the response period was 1400 ms, and in some trials no response was possible within this period. Many premature responses could reflect the initiation of an impulsive response with a high degree of readiness.

#### Number of early responses

A mixed ANOVA with Group (Easy, Hard) and Stimulus Type (RT_FP1.2_, RT_ad_) factors reveal a reliable main effect of Stimulus Type (F (1, 59) = 11.72, p = 0.001, $\eta_{p}^{2}$ = 0.17): The early response rate was lower for RT_ad_ (0.45 ± 0.14%) compared to RT_FP1.2_, (1.77 ± 0.36%). There was no reliable Group effect (F (1, 59) = 1.84, p = 0.181) or Group x Stimulus Type interaction (F (1, 59) = 2.38, p = 0.128).

#### Error rates

We also performed an error-rate analysis using a mixed ANOVA with Group (Easy task, Hard task) and Stimulus (RT_FP0_, RT_ad_, RT_FP1.2_) factors. Significant main effects of Group (F (1, 59) = 8.30, p = 0.006, $\eta_{p}^{2}$ = 0.12) and Stimulus (F (2, 118) = 15.53, p < 0.001, ε = 0.85, $\eta_{p}^{2}$ = 0.21) were found. The Group × Stimulus interaction was nonsignificant (F (2, 118) = 1.80, p = 0.170, $\eta_{p}^{2}$ = 0.03). The proportion of errors in the hard-task group was higher compared to the easy-task group (Easy task: M = 2.86 ± 0.63%, Hard task: M = 5.40 ± 062%). Also, the error rate was higher for RT_FP0_ (6.19 ± 0.74%) compared to RT_FP1.2_ (2.36 ± 0.42%, t (60) = 4.95, p < 0.001) and RT_ad_ in between (3.84 ± 0.59%).

Between-group comparisons for each stimuli category showed that the error rate was higher in the hard-task group compared to the easy-task group for RT_FP0_ (Easy task: 4.17 ± 1.05%, Hard task: 8.23 ± 1.03%, t (59) = 2.75, p = 0.008). The difference between groups was nonsignificant for RT_FP1.2_ (Easy task: 1.50 ± 0.59%, Hard task: 3.23 ± 0.58%, t (59) = 1.56, p = 0.124) and RT_ad_ (Easy task: 2.92 ± 0.84%, Hard task: 4.76 ± 0.83%, t (59) = 2.07, p = 0.042, power = 0.54). Thus, the error rates differences between groups were mainly due to the stimuli with zero constant FP length values.

#### Number of correct responses

Correct responses after trimming were subjected a mixed ANOVA with Group (Easy task, Hard Task) and Stimuli (RT_FP0_, RT_ad_, RT_FP1.2_) factors. Reliable main effects of Group (F (1, 59) = 16.40, p < 0.001, $\eta_{p}^{2}$ = 0.22) and Stimuli (F (2, 118) = 22.15, p < 0.001, ε = 0.88, $\eta_{p}^{2}$ = 0.27) as well as Group × Stimulus interaction (F (2, 118) = 5.07, p = 0.010, ε = 0.88, $\eta_{p}^{2}$ = 0.08) were registered. Group effect indicates that the number of responses in the easy-task group was greater (36.33 ± 0.55) than in the hard-task group (33.19 ± 0.54). Stimulus Type effect indicates that the number of responses for RT_ad_ (36.70 ± 0.39) was greater than RT_FP0_ (34.19 ± 0.51, t (60) = 5.47, p < 0.001) and RT_FP1.2_ (33.40 ± 0.55, t (60) = 6.02, p < 0.001). RT_FP0_ and RT_FP1.2_ did not differ from each other (t (60) = 1.32, p = 0.192).

The reliable Group × Stimulus interaction suggests that the number of responses was higher in the easy-task group compared to the hard-task group in trials with a constant foreperiod RT_FP0_ (Easy task: 36.60 ± 0.72; Hard task: 31.77 ± 0.71; t (59) = 4.75, p < 0.001) and RT_FP1.2_ (Easy task: 34.93 ± 0.79; Hard task: 31.87 ± 0.78; t (59) = 2.76, p = 0.008). The number of trials for RT_ad_ did not differ between groups (Easy task: 37.47 ± 0.56; Hard task: 35.94 ± 0.55; t (59) = 1.95, p = 0.056).

Thus, the number of correct-response trials was lower for RT_FP0_ and RT_FP1.2_ in the hard-task group compared to the easy-task group reflecting that the hard task was more difficult to complete. Also, number of trials with correct responses using the adaptive paradigm was higher compared to the constant paradigm reflecting a tighter congruent coupling between response preparation and response execution processes.

## Discussion

Here, we used an adaptive paradigm in order to estimate the preparatory period duration of motor response in choice reaction time task. The results indicate an increase in response selection duration as a function of enhancing task complexity while using paradigms with constant and adaptive values of the preparatory period duration. Additionally, correlations between the RTs for constant and adaptive FP show that the proposed adaptive paradigm captures the processes that are essential for response preparation and execution. Finally, accuracy data analysis indicates higher accuracy of the adaptive paradigm compared with the constant paradigms tested in this study.

According to the sequential stage model [1, 2] the response to a stimulus includes a sequence of stages of sensory analysis, response selection and the motor execution. Replacing the response selection stage to the post-cuing period reduces the RTs [6–9, 33]. In the present study, the RT for the go stimuli with constant values of the preparatory period lengths confirms these previous findings indicating successful completion of the response selection process during the post-cuing period [6, 7]. Additionally, an increase in response selection complexity in the hard-task group compared with the easy-task group resulted in an RT increase for unprepared responses while the RTs for prepared responses with long FP did not differ between groups. This pattern suggests that response selection process successfully replaced the post-cuing period and confirms the assumption of independence between response selection and response execution processes [34, 35].

In the second set of the analysis we compared RT for constant and adaptive paradigms. We found longer RTs for unprepared responses (constant FP equal zero) than adaptive FP values. This result is partially due to the structural differences between stimuli categories in information flow through the cognitive and motor systems. According to the SSM, an unprepared CRT includes sensory analysis, response selection, and motor execution stages, but an adaptive FP does not include the latter stage. Thus, the difference between unprepared RT and adaptive FP in the current study indicated the duration of motor initiation time, which is 151 ms and this value is little longer than movement initiation time of 131.2 ms for simple responses [33], but the difference between the paradigms should be considered.

The longer RTs for adaptive FP compared to the RTs for long constant FP are likely due to the difference in the preparatory period length–in line with the response preparation effect [5, 6, 33]. We found mean adaptive FP of 544±22 ms significantly shorter, than long constant FP of 1200 ms permits the participants to complete both cognitive and motor preparation processes and activate the corresponding motor program. One can assume, that in the adaptive paradigm, the sum of FP and RT should be comparable to the duration of an unprepared reaction. However, we found that the durations were longer for the adaptive paradigm by 301 and 318 ms for the easy and hard task, respectively. This difference is likely due to the fact that the adaptive paradigm additionally presents an imperative go stimulus, thus, the participant needs to perform the sensory processing and start the motor program.

A recent review by Klapp & Maslovat [25] suggests that following the go stimulus, a delay can be inserted as a response program implementation component. This delay may indicate the participant’s attempt at maximizing the movement accuracy. The proposed adaptive paradigm allows us to examine this period as a part of the RT since participants make fewer errors and premature responses. In contrast, a long constant preparation period produced significantly faster RT, but the cost is in the increase in the number of premature reactions. In this case, the motor system is in the state of a top-down inhibitory control expecting an imperative signal. The predominance of activation over inhibition during this period may trigger a premature response [36]. The proportion of premature responses is an indicator of impulsivity, for instance, children with ADHD show a high proportion of impulsive responses [37].

Also, Maslovat et al [38] reported two separate sub-processes of the reaction preparation process. The first component can be pre-programmed and it includes a sequence of complex actions while the second component includes the temporal organization of the response, which can be partially prepared and partially tied to the motor execution moment. With any explanation (adding a delayed period or preprogrammed by a sub-process), this RT increase for adaptive FP compared with long constant FP does not reflect a selection process since it does not interact with the task complexity. At the same time, this component is likely to be cognitive since it is not rigidly tied to the response execution, and it can be moved to the preparatory period in proper conditions. The nature of this component is not entirely clear and it requires further investigation.

Share processes also could be proposed for the constant and adaptive paradigms. First, our analysis showed a nonsignificant effect of the FP Type factor to the Group and FP Location suggesting that the proposed adaptive paradigm captured the same basic components of the cognitive and motor response regulation as the constant FP values paradigm. Second, our correlation analysis provided additional evidence regarding the paradigms’ common aspects. Strong positive correlations were found between all variables for both participant groups, indicating shared variance for both paradigms. However, correlations were smaller for constant FPs in hard-task group, than for constant FPs in easy-task group. Thus, the complexity of the task reduced the correlation between RTs. The results may follow the fact that constant FPs include additional processes that reduce coupling, including different levels of complexity in response selection and response expectation mechanisms for long FPs. Also, the association between adaptive FP and adaptive RT was stronger than the association between RTs with constant FPs. An adaptive FP includes fewer additional processes, and a response execution follows response selection in timely congruent fashion. This difference could be explained by the paradigm itself since the link between FP and RT for adaptive paradigm was pre-programmed. However, FP was calculated for the *n+1* trial and the relationship between FP and RT should be rather negative than positive, according to the preparation effect. This relationship was revealed in constant FPs whereby a longer preparation time results in a shorter RT. However, the results of the inter-trial correlations within participants for the adaptive FP and RT were close to zero. These results complement existing findings for constant FP and indicate a degree of independence between the processes of response preparation and execution [2, 34, 35]. Similar results were reported in the study by Servant et al. [24]. Using a random dot motion task, the study found strong positive between-participant correlations (r>0.95) between the duration of the premotor period and the duration of the motor period as well as low values of the within-subject correlations (r<0.13) at different levels of complexity of the perceptual task.

Additionally we analyzed accuracy data. Constant FP values were associated with a lower accuracy compared to the adaptive paradigm. For an unprepared response, the selection process produced a larger proportion of missed and erroneous responses. On the other hand, a large proportion of premature responses was found for prepared responses with long constant FP. These results are in line with the Prepared reflex approach [12, 26], which states that a voluntary response includes both the voluntary component, more associated with the response selection, and the automatic component, closer tied to the response implementation. While a response is prepared with a high degree of certainty, the response execution can be triggered unexpectedly, resulting in premature or early responses. The results of our error-rate analysis suggest that the adaptive paradigm did not replicate the patterns observed in the constant FP values. Paradoxically, performance in a more complex adaptive paradigm required monitoring of two successive stimuli, leading to more accurate participant’s responses. However, these results can be interpreted as a general effect of the foreperiod length since the adaptive foreperiod values lie between the constant FPs. Therefore, the position of the mean duration of the adaptive FP on the dependency curve of RT on FP values remains unclear.

We conclude that our adaptive paradigm offers a number of features useful for future research. First, the constant FP values paradigm uses the preparatory period duration as a testing method and it is incapable of calculating the individual duration of the response selection process, compared with the adaptive paradigm. Second, in the constant FP values paradigm response selection is investigated by comparison simple and complex selection conditions. In this case, at least two stimuli categories are necessary. However, in the adaptive paradigm, one can use simple RT for a single stimulus category and get the duration of its preparatory period. Third, the adaptive paradigm does not include an additional process of expecting the go signal while the selection process is not influenced by the additional decision process aimed at estimating selection completeness as in self-select paradigm [15, 16, 17]. Using the adaptive paradigm, the FP duration is calculated automatically, and the result is therefore less arbitrary and more objective. Forth, classical DDM [18] and LBA [21] models are widely used to analyze decision-making processes. However, these parameters depend on the selected distribution type [19] calculated after the completion of the task and required additional verification [20]. In the adaptive paradigm, FP values are obtained online and allow manipulation parameters during the task execution (for instance, hardening of criteria or response bias). Also, the calculation is quite simple, allowing the paradigm to be used in a wide range of studies.

Our study has several limitations.

First, although the adaptive paradigm assumes a decrease in the variability of the RT and the FP duration, the results showed no difference between adaptive FP and RT for short constant FP as well as between adaptive RT and RT for long constant FP. These results could be based on at least two factors that increase the variability of responses. The first one is that there were few trials in each condition. On average, a slightly more than 30 trials may not be enough to find a steady motor state for each participant, and a significant increase in the number of trials increase the sustainability of results. However, the weight of learning factor is increases, making it difficult to interpret the results. Other factor influencing the variability of response duration is the randomized presentation of stimuli. A study by Van der Lubbe et al [39] found the effect of the FP duration in *n-1* trial on the RT in *n* trial predominantly with short FP. Thus, random presentation of trials with prepared and unprepared responses increases the variability in the RT and FP length in adaptive paradigm. The use of the block design of stimulus presentation could reduce the variability of responses.

Second, small number of trials and mixing different stimulus types does not allow to calculate DDM parameters in order to investigate its relationships with the parameters of adaptive FP and RT. Considering the first limitation block design with alternating adaptive FP blocks and CRT blocks allows to calculate both DDM and adaptive FP parameters.

Third, the proposed algorithm for calculating the preparatory period in the *n+1* trial based on the RT in the trial *n* is linear in the present study. Updating of the FP length in the adaptive paradigm is similar to the Bayesian statistic of posterior distribution based on the obtained data. The use of the Bayesian method for updating the duration of the reaction preparation period based on the obtained RT data is also a possible solution for finding the optimal value of the latent parameter for the response time.

Forth, only the boundary conditions of a completely unprepared and fully prepared responses were used in the current study. The effects reported in the present study can result from the general differences in the FP duration rather than from the adaptive algorithm itself. To address this issue, the future research will need to use stimuli presentation with constant preparatory period durations (for example, 100, 200, 300, 400, 500, 600 ms). This, however, will substantially increase the experimental session duration and may affect the duration of the adaptive preparatory period as well as the corresponding reaction time values. Nevertheless, such a paradigm will allow to analyze the adaptive paradigm data on a dependency curve between the RT and the FP lengths. Based on our findings, we can assume that it could be located in the point of the maximal inflation of the curve. In addition, the use of a constant FP comparable in duration to the adaptive FP as an additional control condition is also problematic for several reasons. We proposed a new paradigm, for which there are no known normative data on the mean length of the adaptive FP. Also, we observed a significant variability of the individual FP duration values, so it would be incorrect to use one constant value for the duration of the preparatory period.

To conclude, we report a validation study for a novel paradigm of determining the individual length of the preparatory period in the choice RT task. Our data demonstrate a strong correlation with the results with constant lengths of the preparatory period in easy and difficult tasks. However, our method also has important advantages, and it creates an opportunity to use the adaptive method in a wide range of fundamental research and clinical studies.

### Ethics approval

Approval was obtained from the ethics committee of the Higher School of Economics. The procedures used in this study adhere to the tenets of the Declaration of Helsinki.
