# Correction: An adaptive paradigm for detecting the individual duration of the preparatory period in the choice reaction time task

**DOI:** 10.1371/journal.pone.0315215

**Published:** 2024-12-04

**Authors:** Gurgen Soghoyan, Vladislav Aksiotis, Anna Rusinova, Andriy Myachykov, Alexey Tumyalis

There are errors in the Funding statement. The correct Funding statement is as follows: This work is supported by the Center for Bioelectric Interfaces NRU HSE, RF Government Grant, AG. No. 075-15-2021-624.
